# The Impact of COVID-19 on Interventional Radiology Services in the UK

**DOI:** 10.1007/s00270-020-02692-2

**Published:** 2020-11-03

**Authors:** Jim Zhong, Anubhav Datta, Thomas Gordon, Sophie Adams, Tianyu Guo, Mazin Abdelaziz, Fraser Barbour, Ebrahim Palkhi, Pratik Adusumilli, Mohammed Oomerjee, Edward Lake, Paul Walker

**Affiliations:** 1 Department of Diagnostic and Interventional Radiology, Leeds Teaching Hospitals NHS Trust, Leeds, LS9 7TF UK; 2 Department of Diagnostic and Interventional Radiology, The Christie NHS Foundation Trust/ University of Manchester, Manchester, UK; 3 Department of Diagnostic and Interventional Radiology, Manchester University NHS Foundation Trust, Manchester, UK; 4 Department of Diagnostic and Interventional Radiology, NHS Lothian, Edinburgh, Scotland; 5 Department of Diagnostic and Interventional Radiology, NHS Greater Glasgow and Clyde, Glasgow, Scotland; 6 Department of Diagnostic and Interventional Radiology, The Mid Yorkshire Hospitals NHS Trust, Wakefield, UK; 7 Department of Diagnostic and Interventional Radiology, Bradford Teaching Hospitals NHS Foundation Trust, Bradford, UK

**Keywords:** COVID-19, Pandemic, Interventional radiology, Services, Workload, Acute, Elective

## Abstract

**Introduction:**

The coronavirus disease 2019 (COVID-19) has created unprecedented challenges on the healthcare system. The aim of this multi-centre study was to measure the impact of COVID-19 on IR services in the UK.

**Material and Methods:**

Retrospective cross-sectional study of IR practice in six UK centres during the COVID-19 pandemic was carried out. All therapeutic IR procedures were identified using the respective hospital radiology information systems and COVID-19 status found on the hospital patient record systems. The total number of therapeutic IR procedures was recorded over two time periods, 25/03/2019–21/04/2019 (control group) and 30/03/2020–26/04/2020 (COVID-19 group). The data points collected were: procedure type, aerosol-generating nature, acute or elective case, modality used, in- or out-of-hours case and whether the procedure was done at the bedside (portable).

**Results:**

A 31% decrease in overall number of IR procedures was observed during COVID-19 compared to the control group (1363 cases vs 942 cases); however, the acute work decreased by only 0.5%. An increase in out-of-hours work by 10% was observed. COVID-19 was suspected or laboratory proved in 9.9% of cases (*n* = 93), and 15% of total cases (*n* = 141) were classed as aerosol-generating procedures. A 66% rise in cholecystostomy was noted during COVID-19. Image-guided ablation, IVC filters, aortic stent grafting and visceral vascular stenting had the greatest % decreases in practice during COVID-19, with 91.7%, 83.3%, 80.8% and 80.2% decreases, respectively.

**Conclusion:**

During the global pandemic, IR has continued to provide emergency and elective treatment highlighting the adaptability of IR in supporting other specialties.

**Electronic supplementary material:**

The online version of this article (10.1007/s00270-020-02692-2) contains supplementary material, which is available to authorized users.

## Introduction

The novel coronavirus disease 2019 (COVID-19) was officially declared a pandemic by the World Health Organization on 11 March 2020. During the subsequent weeks, the rising number of new COVID-19 cases and hospital admissions in the UK resulted in increased pressures on the national health service (NHS). In an attempt to tackle these issues, the UK government issued a national lockdown on 23 March 2020. Despite these strict measures, cases continued to rise and COVID-19 death rates peaked in mid-April [1]. As of the 23 July 2020, the UK had the highest recorded number of deaths within Europe, with over 50,000 COVID-19-related deaths [1].

Like other healthcare systems around the world that have quickly adapted their acute services, NHS hospitals swiftly responded to the pandemic, by halting elective services, to increase capacity for the expected surge in patients requiring urgent respiratory support. COVID-19 has brought new challenges for interventional radiology (IR), both in terms of workflow and IR preparedness; however, IR has continued to provide acute and emergency treatments due to the unique value that IR has to offer in terms of adaptability and offering minimally invasive procures (MIPs), the majority of which are done under local anaesthetic which are not aerosol-generating procedures (AGPs) [2, 3].

The aim of this multi-centre study was to review the impact of COVID-19 on IR services in terms of workload and case mix.

## Methods

This study was a retrospective multi-centre cross-sectional study of IR practice during the COVID-19 pandemic. No formal ethical approval was required as these were audit data. The study was registered and approved within each Trust’s audit department. No identifiable patient data were kept or reported. This study required no internal or external funding. The authors have no conflicts of interest to declare.

All therapeutic IR procedures were identified using the respective hospital radiology information systems and COVID-19 status found on the hospital patient record systems.

The number of IR procedures was recorded over two time periods, 25/03/2019–21/04/2019 (control group) and 30/03/2020–26/04/2020 (COVID-19 group).

The study period for 2020 was selected to encompass the peak in COVID-19 cases throughout the UK and capture the subsequent effect on the provision of IR services. The same 4-week interval in 2019 was captured which formed a control group for comparing the impact of COVID-19.

This was replicated in six NHS trusts and health boards (Leeds, Manchester, Pinderfields, Bradford, Edinburgh and Glasgow) to measure the variation in baseline workload and subsequent impact from COVID-19.

For all recorded IR cases, we included type of procedure (categories provided in supplementary material), acute/emergency or time-critical elective case, modality used, out of hours or in hours and whether the procedure was done at the bedside (portable) guidance for what constitutes as AGPs was from Sect. 5 of the joint Cardiovascular and Interventional Radiological Society of Europe (CIRSE) and Asia Pacific Society of Cardiovascular and Interventional Radiology (APSCVIR) checklist for COVID-19 [4]. This includes procedures that involve airway suctioning, access via nasal/oral routes and procedures which may induce coughing and subsequent aerosolisation.

Time-critical elective procedures were defined as an intervention that was planned or booked in advance of the routine admission to hospital [5]. These remained time-critical cases including intervention for critical limb ischaemia and transarterial chemoembolisation for cancer, as cancellations or significant delays to such procedures would have severe knock-on effects to overall health and quality of life, and lead to unnecessary deaths.

Acute emergency procedures were defined as inpatient cases done for urgent, possibly life- or limb-threatening conditions, for example, Neuro-IR/stroke intervention, aortic stent grafting for ruptured aneurysm, embolisation for intra-abdominal bleeding, peripheral intervention for acute limb ischaemia, image-guided drainage for infective collections, pneumothoraces or tense ascites.

For the 2020 (COVID-19) group, we included COVID-19 status at the time of procedure which was split into three categories: confirmed COVID-19 case if polymerase chain reaction (PCR) test positive, suspected COVID-19 based on clinical or imaging evidence (even if PCR test negative) or not suspected with or without COVID-19 negative PCR test.

## Results

**Fig. 1:**
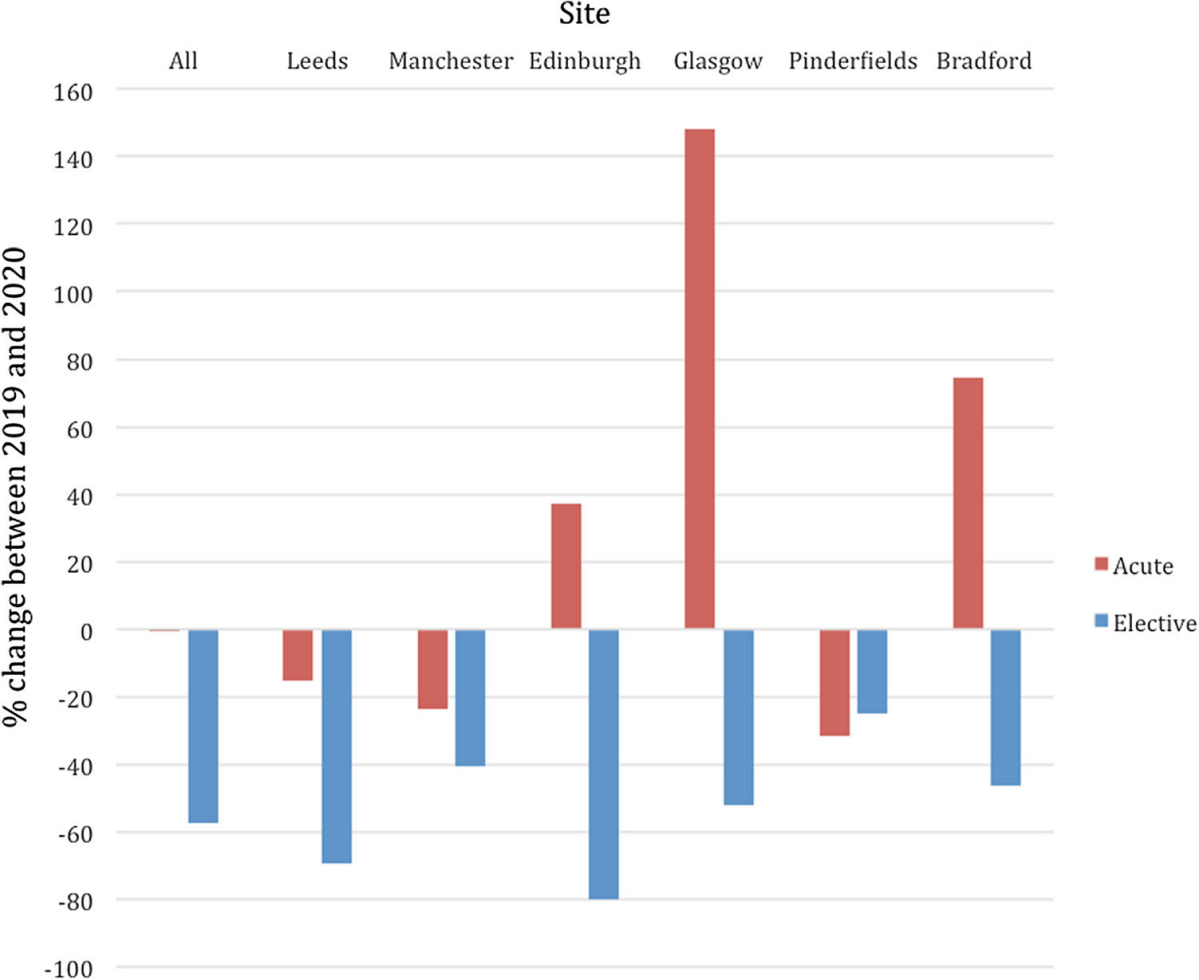
Graph demonstrating all sites and site-specific percentage changes in IR workload. Acute work is demonstrated by the orange bar charts, and elective work is demonstrated by the blue bar charts. Whilst all sites report a decrease in elective workload, three sites have noticed a corresponding increase in acute cases

The absolute number and types of cases during the pre-COVID-19 period and COVID-19 period are shown in Table 1. There has been a 31% decrease in the overall IR caseload when the two time periods are compared (1363 cases vs 942 cases), and every site reports a decrease in workload (ranging between a 10% decrease and a 40% decrease). Results of subgroup analyses are presented as changes in acute workload and changes in elective workload (Fig. 1).

**Table 1:** The number and types of IR cases for the pre-COVID-19 and COVID-19 period are shown including the percentage (%) change. Aerosol-generating procedures (AGPs) are shown in bold.

	IR case mix		
	Pre-COVID-19 (2019 data) *n* = 1363	COVID-19 (2020 data) *n* = 942	% Change
Acute/elective	637/726	634/308	−0.5%/−57.6%
In/out of hours	1228/135	793/149	−35.4%/ + 10.4%
Portable procedures	6	5	−16.7%
Fluoroscopy-guided	1123	748	−33.3%
Ultrasound-guided	212	181	−14.6%
CT-guided	28	13	−53.3%
Type of procedure			
Abdominal drainage	154	141	−8.4%
Nephrostomy/ureteric stenting	133	111	−16.5%
**Chest drain**	**54**	**42**	−**22.2%**
Gallbladder drain	15	25	+ 66.7%
**Feeding tube/GI stenting**	**125**	**97**	−**22.4%**
PTC/biliary stenting	34	25	−26.5%
Image-guided ablation	24	2	−91.7%
Vascular access	237	185	−21.9%
Peripheral vascular intervention/stenting	195	145	−25.6%
**Thoracic intervention/embolisation**	**12**	**2**	−**83.3%**
Abdominal embolisation	108	46	−57.4%
IVC filter	30	5	−83.3%
Visceral vascular stenting	86	17	−80.2%
Fistuloplasty	86	61	−29.1%
TEVAR/EVAR	26	5	−80.8%
Neuro-IR/stroke intervention	44	33	−25.0%

Key: *CT* computed tomography, *PTC* percutaneous transhepatic cholangiography, *IVC* inferior vena cava, *TEVAR* thoracic endovascular aortic repair, *EVAR* endovascular aortic repair

The impact of COVID-19 on acute IR work is site variable. Three out of the six sites report a decrease in workload, whereas the other 50% have performed more cases in the acute setting when compared to the pre-COVID-19 time period. The resulting effect on all site average is a 0.5% decrease in acute work. On the other hand, the trend for impact on elective work was a uniform decrease across all sites with an overall decrease of 58% (range 25%–80%). CT-guided procedures had the greatest decrease in workload (53.3%) compared to fluoroscopy-guided (33.3%) and ultrasound-guided (14.6%) procedures.

**Fig. 2:**
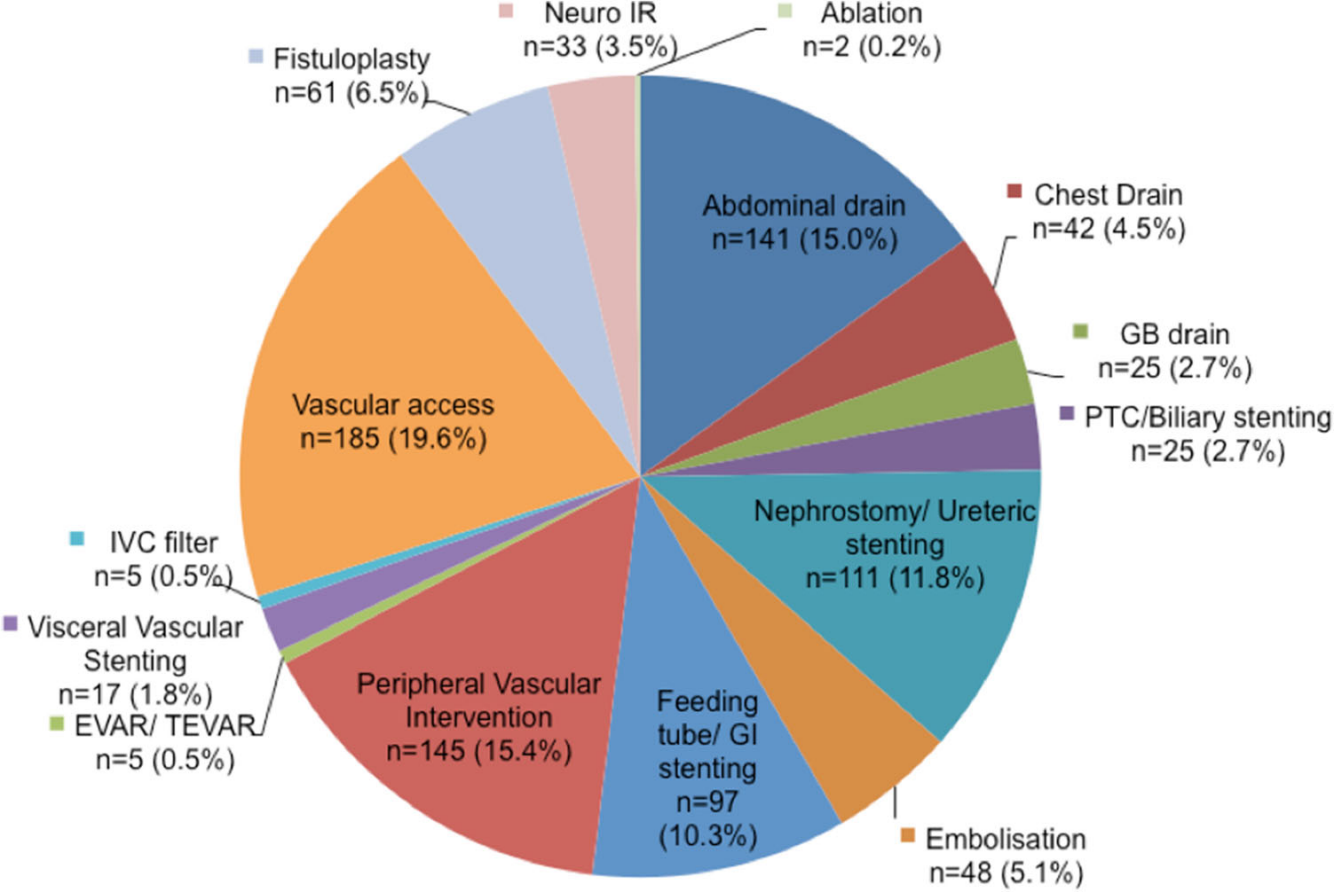
IR case mix during COVID-19 pandemic across all six centres

The IR case mix during the COVID-19 pandemic across the six centres is highlighted in Fig. 2.

The most common procedures performed during the COVID-19 period were vascular access, abdominal drainages, peripheral vascular intervention and nephrostomies ± ureteric stenting. The areas of IR that had the greatest decrease in activity during the pandemic were image-guided ablation (91.7% reduction), placement of IVC filters (83.3% reduction), aortic stent grafting (80.8% reduction) and visceral vascular stenting (80.2% reduction).

The total number of gallbladder drains performed increased by 67% during the COVID-19 pandemic. The out-of-hours work also increased by 10.4% during the pandemic.

During the 2020 study period, there were 22 IR cases (2.3%) of confirmed COVID-19. An additional 71 cases were treated as suspected COVID-19 (7.5%). No IR physicians or allied healthcare professionals were directly infected from the cases of confirmed or suspected COVID-19.

## Discussion

The COVID-19 pandemic had a profound immediate impact on IR workload. We report a 31% decrease in the number of cases performed in the first 4 weeks of the COVID-19 lockdown when compared to an equivalent time period from 2019. The six sites reported a range of decreasing workload from 10 to 40%. Existing published data have measured the impact of COVID-19 on other hospital specialities, with a focus on acute and elective subgroups [6–12]. We have adopted the same approach to ensure consistency in reporting.

A major aim of this study was to quantify, and attempt to qualify, the impact of the national COVID-19 safety measures within the hospitals delivering the care. The procedure-related risk of the COVID-19 infection depends on many factors, which can be broadly considered as operator, patient and environment. We highlighted the COVID-19 status of the patient and AGPs as two of the most important surrogate markers for procedure-related exposure. It is important to note that infection control remains a continuously evolving topic. We have used the risk standards during the period of observation to maintain the context of perceived and actual risk. For example, a major NHS Foundation Trust at the time instructed staff to only perform cardiopulmonary resuscitation on cardiac arrest patients if they were in the emergency department and all staff members were wearing PPE [13]. In our 2020 results, 9.9% of the total cases (*n* = 93) were either suspected or laboratory proven COVID-19, and 15% of total cases (*n* = 141) were classified as AGPs. As radiology is often perceived as a non-patient facing speciality, the staff can be miscategorised as relatively low risk from COVID-19 when compared to other hospital environments. However, this study clearly highlights the significant risk to staff members who work within the IR sub-speciality and challenges this notion, however thankfully there were no recorded cases of infection amongst IR team members from the confirmed or suspected cases. Furthermore, this represents a significant impact on day-to-day practice as these cases required extra planning, resources and safety. Complete personal protective equipment (PPE) was worn by all the staff involved in the cases which placed a greater emphasis on clear communication and team-work during the procedure.

National figures reported by Public Health England state 2.11 million Accident and Emergency (A&E) attendances in April 2019 compared to only 916,000 attendances in 2020, which represents some of the lowest figures in recent times [6, 14]. A decrease in trauma admissions by 54% has also been observed in the UK [7, 8]. Similarly, a survey of 43 UK centres reports that 36% of cardiac cath laboratories were also closed during the UK COVID-19 lockdown [9]. Whilst it is uncommon for interventional radiology to have acute referrals straight from A&E, we anticipate that such a dramatic decrease will have impacted the workload. Major changes in patients health-seeking behaviour have also reduced the routine referrals from GPs as patients were asked to only present if they had major or urgent concerns [10].

Our results show a site-specific variability in the impact on acute work. We believe the variation in reported acute work is due to several reasons despite the same national guidance given to all the sites. The sites within our study vary in size, and therefore, the IR departments will have a different volume of baseline work, available staff and capacity to run a ‘COVID-19 appropriate’ rota. It is also important to appreciate that the impact of COVID-19 in the UK is geography dependent. These factors generate the need to develop locally specific policies to ensure ongoing safety. Furthermore, acute work in IR is heavily influenced by workload in other clinical specialities, and we often provide complementary patient care. Due to the safety concerns of viral transmission secondary to AGPs, MIPs were preferred in managing acutely unwell patients. This may explain why acute IR services have remained busy and not seen the same dramatic decreases seen elsewhere, i.e. a negligible 0.5% decrease in acute work between the two time periods. In the context of an overall decrease in emergency cases across the NHS, one assumption would be that the proportional work done by IR has increased with the finding of a 10% rise in out-of-hours work unsurprising. Uncertain working conditions require clinicians to be flexible in their approach, and IR services in this study have been able to adapt and successfully deliver front-line patient care, likely attributable to efficient methods of workflow reconfiguration and availability to treat patients. Similar trends have been observed in the US, where IR procedural volumes have decreased by a significantly smaller amount compared to other surgical, cardiac and endoscopy services [15].

Upper gastrointestinal endoscopy procedures are classified as AGPs with increased risk of virus transmission. This led to consensus guidance from the British Society of Gastroenterology, American College of Gastroenterology, European Society of Gastrointestinal Endoscopy and Asian Pacific Society for Digestive Endoscopy that elective endoscopies should be suspended [11]. This resulted in a 90% reduction in the number of endoscopies undertaken in April 2020 in the UK, compared with the first 3 months of 2020, based on data from the UK National Endoscopy Database [12, 16]. IR availability has allowed for the continued insertion of feeding tubes or biliary intervention with fluoroscopy, which in certain centres may have undergone the endoscopy route first-line, with smaller decreases in cases observed (22–27%).

Surgical practice has also been greatly affected during the pandemic with the British Intercollegiate General Surgery Guidance on COVID-19 stating that ‘whenever non-operative management is possible (such as for early appendicitis and acute cholecystitis), this should be implemented’ [17]. The theoretical risk of viral transmission from creating a pneumoperitoneum required for both laparoscopic and robotic surgeries has resulted in a subsequent decrease in such activity [18]. The subsequent decrease in laparoscopic cholecystectomies performed may explain the current study’s finding of an increase in the number of cholecystostomies undertaken by IR, given this is an alternative minimally invasive therapy which is non-aerosol generating that allows for treatment of acute cholecystitis, if conservative management with antibiotics fails. Before COVID-19, cholecystostomy was usually only indicated in patients who were not eligible for surgery, supported by the CHOCOLATE randomised clinical trial [19]. During COVID-19, with less availability of laparoscopic surgery, the threshold to intervene with cholecystostomy was likely lower.

In contrast to the acute setting, there was a uniform decrease in elective cases performed as per the national guidance given. Remarkably, however, all sites continued providing elective services, used to help patients requiring time-critical treatment, or those waiting longer than expected due to treatment delays, including renal dialysis and fistula work. The proposed aim was to reduce the immediate backlog of patients as the NHS headed into uncertain times. The ability to deliver routine services amidst a respiratory viral pandemic is essential and intrinsic to IR, particularly important as a second COVID-19 wave is looming, with our data highlighting areas where IR can step in to support other acute services [20]. This further underpins the need for investment and recruitment into IR. An example from our experience is that a dedicated radiology admission day unit (RADU) can help isolate symptom-free outpatients from the main hospital. This therefore further reduces the risk of transmission and helps maintain patient confidence to attend their appointment.

The limitations of this study are the retrospective design. The study includes relatively small numbers of certain IR procedures; however, the multi-centre design allowed for a wider geographic area to be sampled to give a more accurate representation of the true impact of COVID-19 on IR services. Only the peak period of the COVID-19 pandemic was sampled (4 weeks) rather than the entire UK lockdown period.

## Conclusion

IR has continued to provide emergency treatment whilst maintaining some time-critical elective services throughout the pandemic highlighting the adaptability of IR in supporting many specialties. The unique skill set of IRs allows them to play a key role during COVID-19 and future system-wide crises.

## Electronic supplementary material

Below is the link to the electronic supplementary material.

Supplementary file1 (DOCX 103 kb)

## References

[1] Office for National Statistics; Deaths registered weekly in England and Wales, provisional: week ending 10 July 2020 [Internet]. 2020.

[2] Pua U, Wong D (2020). What is needed to make interventional radiology ready for COVID-19? lessons from SARS-CoV epidemic. Korean J Radiol [Internet].

[3] Da Zhuang K, Tan BS, Tan BH, Too CW, Tay KH (2020). Old threat, new enemy: is your interventional radiology service ready for the coronavirus disease 2019?. Cardiovasc Intervent Radiol [Internet].

[4] Cardiovascular and Interventional Society of Europe; Asia Pacific Society of Cardiovascular and Interventional Radiology; Joint CIRSE-APSCVIR checklist to prepare IR departments for COVID 19. 2020. https://www.cirse.org/wpcontent/uploads/2020/04/cirse_APSCVIR_Checklist_COVID19_prod.pdf. Accessed 29 Oct 2020.

[5] National Confidential Enquiry into Perioperative Deaths (NCEPOD). The NCEPOD classification of intervention. 2004. https://www.ncepod.org.uk/classification.html. Accessed 29 Oct 2020.

[6] NHS England. A&E Attendances and Emergency Admissions 2019-2020. 2019. https://www.england.nhs.uk/statistics/statistical-work-areas/ae-waitingtimes-and-activity/ae-attendances-and-emergency-admissions-2019-20/. Accessed 29 Oct 2020.

[7] Hampton M, Clark M, Baxter I, Stevens R, Flatt E, Murray J (2020). The effects of a UK lockdown on orthopaedic trauma admissions and surgical cases. Bone Jt Open.

[8] Sugrue CM, Sullivan P (2020). The effect of the ongoing COVID-19 nationwide lockdown on plastic surgery trauma caseload?. J Plast Reconstr Aesthetic Surg [Internet].

[9] Adlan A, Lim V, Dhillon G, Kurdi H, Doolub G, Elamin N (2020). Impact of COVID-19 on primary percutaneous coronary intervention centres in the UK: a survey. Br J Cardiol [Internet].

[10] Jones D, Neal RD, Duffy SRG, Scott SE, Whitaker KL, Brain K (2020). Impact of the COVID-19 pandemic on the symptomatic diagnosis of cancer: the view from primary care. Lancet Oncol [Internet].

[11] Chiu PWY, Ng SC, Inoue H, Reddy DN, Ling HuE, Cho JY (2020). Practice of endoscopy during COVID-19 pandemic: position statements of the Asian Pacific Society for Digestive Endoscopy (APSDE-COVID statements). Gut [Internet].

[12] Richards M, Anderson M, Carter P, Ebert BL, Mossialos E (2020). The impact of the COVID-19 pandemic on cancer care. Nat Cancer [Internet].

[13] Mahase E, Kmietowicz Z (2020). Covid-19: doctors are told not to perform CPR on patients in cardiac arrest. BMJ [Internet].

[14] NHS England. A&E Attendances and Emergency Admissions 2020-21. 2020. https://www.england.nhs.uk/statistics/statistical-work-areas/ae-waitingtimes-and-activity/ae-attendances-and-emergency-admissions-2020-21/. Accessed 29 Oct 2020.

[15] Patel MV, Ahmed O, Hennemeyer C, Hatchett S, Sacramento M, Funaki B. IR is an operational and financial hedge for hospitals during COVID-19. J Vasc Interv Radiol. 2020;31(10):1724–1726. 10.1016/j.jvir.2020.07.019. 10.1016/j.jvir.2020.07.019 PMC7377721 32943298

[16] Joint Advisory Group on GI Endoscopy. National Endoscopy Database. https://www.ned.jets.nhs.uk. Accessed 10 Oct 2020.

[17] Royal College of Surgeons of England; Updated Intercollegiate General Surgery Guidance on COVID-19. 2020. https://www.rcseng.ac.uk/coronavirus/joint-guidance-for-surgeons-v2/. Accessed 29 Oct 2020.

[18] Vigneswaran Y, Prachand VN, Posner MC, Matthews JB, Hussain M. What is the appropriate use of laparoscopy over open procedures in the current COVID-19 climate?. J Gastrointest Surg. 2020;24(7):1686–1691. 10.1007/s11605-020-04592-9. 10.1007/s11605-020-04592-9 PMC7153352 32285338

[19] Loozen CS, Van Santvoort HC, Van Duijvendijk P, Besselink MG, Gouma DJ, Nieuwenhuijzen GA (2018). Laparoscopic cholecystectomy versus percutaneous catheter drainage for acute cholecystitis in high risk patients (CHOCOLATE): multicentre randomised clinical trial. BMJ [Internet].

[20] Middleton J, Lopes H, Michelson K, Reid J (2020). Planning for a second wave pandemic of COVID-19 and planning for winter: a statement from the association of schools of public health in the European region. Int J Public Health [Internet].

