# Supplementary material for: The Impact of COVID-19 on Interventional Radiology Services in the UK
Source: Cardiovasc Intervent Radiol. 2020 Nov 3;44(1):134–40. doi: 10.1007/s00270-020-02692-2 (PMC7609351; doi:10.1007/s00270-020-02692-2)
Supplement: Supplementary file 1 — Supplementary file1 (DOCX 103 kb) [file 270_2020_2692_MOESM1_ESM.docx]

**Interventional Radiology Procedures included:**

- Abdominal Drainage
  - Ascitic drain
  - CT/ US-guided abdominal drainage for collections
  - Liver/ splenic abscess drainage
  - Suprapubic catheter
- Nephrostomy/ Ureteric stenting
- Chest Drain
  - For pneumothorax or effusion/ collection
- Gallbladder drain
- Feeding Tube/ GI stenting
  - Fluoroscopically guided nastro-gastric and naso-jejunal tube
  - Radiologically inserted gastrostomy tube (RIG)
  - Upper and lower GI stenting
- Percutaneous transhepatic cholangiography (PTC) / Biliary stenting
- Image-guided ablation
  - Radio-frequency ablation/ Microwave ablation/ Cryoablation/ Irreversible electroporation
- Vascular Access
  - Central or peripheral line insertion
- Peripheral vascular intervention/ stenting
  - Iliac/ Femoral/ Popliteal/ Crural angioplasty
- Thoracic embolisation
  - Bronchial artery embolisation
  - Pulmonary arteriovenous malformations
  - Catheter-directed thrombolysis for pulmonary embolism
- Abdominal embolisation
  - Transarterial chemoembolisation
  - Portal vein embolisation
  - Trauma embolisation for bleeding
  - Visceral aneurysm embolisation
  - Venous embolisation for testicular varicocele or pelvic congestion syndrome
- IVC filter
- Visceral vascular stenting
  - Renal artery angioplasty/ stenting for native kidney or transplant kidney
  - Hepatic artery angioplasty/ stenting (e.g. post-transplant)
  - Coeliac or Superior mesenteric artery angioplasty/ stenting
- Fistuloplasty
  - Including central venous angioplasty related to fistula and catheter directed thrombolysis for thrombosed fistula
- Thoracic endovascular aortic repair (TEVAR) or endovascular aortic repair (EVAR)
- Neuro IR/ Stroke intervention
  - Mechanical thrombectomy
  - Aneurysm coiling
  - Carotid artery stenting
  - Arteriovenous malformation (AVM) embolisation
